# Psychometric evaluation of the Brief Multidimensional Students' Life Satisfaction Scale (BMSLSS) in Chilean early adolescents: an Item Response Theory analysis

**DOI:** 10.3389/fpsyg.2025.1638017

**Published:** 2025-09-17

**Authors:** René Gempp, Mònica González-Carrasco

**Affiliations:** ^1^Facultad de Administración y Economía, Universidad Diego Portales, Santiago, Chile; ^2^Research Institute on Quality of Life (IRQV), Universitat de Girona, Girona, Spain

**Keywords:** Brief Multidimensional Students' Life Satisfaction Scale (BMSLSS), Item Response Theory (IRT), Graded Response Model (GRM), differential item functioning (DIF), MIMIC model, life satisfaction, early adolescents, Chile

## Abstract

**Introduction:**

Life satisfaction is a core component of subjective wellbeing (SWB) that contributes significantly to child and adolescent development and outcomes. The *Brief Multidimensional Students' Life Satisfaction Scale* (BMSLSS) is one of the most popular measures of life satisfaction in childhood and adolescence. Most studies have validated this scale using classical psychometric methods. The purpose of this study was to thoroughly investigate the psychometric properties of the BMSLSS using Item Response Theory, in Chile.

**Methods:**

We used Samejima's Graded Response Model (GRM) to investigate the item response functions, item and total precision, as well as gender and age differential item functioning (DIF) of the BMSLSS in a nationwide cross-sectional sample of *n* = 5,619 Chilean early adolescents (49.2% girls), aged 10, 11, and 12 years (46.13%, 44.99%, and 8.88% respectively).

**Results:**

Conventional psychometric analyses, including item-rest correlations, internal consistency, and factor analysis, indicated good overall functioning of the BMSLSS. However, the IRT analysis revealed important nuances in the scale's performance. Specifically, results showed redundancy among lower response categories and insufficient discrimination at the upper end of the life satisfaction spectrum. The analysis also detected measurement non-invariance for some items across gender and age.

**Discussion:**

These findings suggest that while the BMSLSS demonstrates adequate overall performance, the results advise reviewing the items' response options and exercising caution when interpreting high scores. Implications and future directions of research are discussed.

## Introduction

### The importance of subjective wellbeing in childhood and adolescence

In recent decades, the study of subjective wellbeing (SWB) in childhood and adolescence has received growing attention within psychology and the social sciences (Casas et al., 2014, 2018; Jonsson et al., 2017) due to the compelling evidence showing that children's and adolescent's self-evaluation of their own lives – both overall and with specific domains such as family, friends, and leisure time—plays a pivotal role in their development (Ben-Arieh et al., 2014). Research has consistently shown that SWB influences physical, social, psychological, and academic functioning (Casas et al., 2014, 2018; Jonsson et al., 2017).

There are two major theoretical conceptualizations of SWB: the eudaimonic and hedonic approaches (see Ryan and Deci, 2001, for a review). The hedonic tradition conceptualizes SWB as comprising both cognitive and affective components: life satisfaction represents the cognitive component, while positive and negative emotions constitute the affective dimension, reflecting the so-called tripartite structure theory of SWB (Arthaud-Day et al., 2005; Diener, 1984; Metler and Busseri, 2017). Furthermore, within the cognitive component of SWB, researchers distinguish between global life satisfaction and domain-specific satisfaction (Diener, 1984; Diener et al., 1999). While global life satisfaction reflects an overall evaluation of one's life, domain satisfaction focuses on specific life areas such as family, friends, school, self, and living environment. The relationship between these components has evolved from simple aggregation models to more complex conceptualizations, including the recent four-factor model of SWB that separates global and domain satisfactions as distinct but related factors (González-Carrasco and Casas, 2024).

Research has consistently demonstrated that SWB in youth is associated with critical developmental outcomes. Higher levels of SWB in childhood and adolescence predict positive psychological outcomes including effective stress coping, resilience, academic achievement, and psychological adjustment (Heffner and Antaramian, 2016; Jiang et al., 2016, 2019; Lewis et al., 2011; Ng et al., 2015; Sun and Shek, 2010). Moreover, adolescent SWB correlates with positive future orientation, capacity for adult life planning, and serves as a protective factor for adult mental health (Li et al., 2023; Otto et al., 2021).

Conversely, low SWB during childhood and adolescence is associated with adverse developmental outcomes. These include low self-esteem, low self-efficacy, high-risk behaviors, bullying victimization, depressive symptoms, delinquent behaviors, substance abuse, dating violence victimization experiences, and unhealthy lifestyle behaviors, and they are associated with a higher risk of mental health problems in adulthood (Blood et al., 2011; Cava et al., 2021; Jovanović, 2022; Jung and Choi, 2017; Mohamad et al., 2018; Moksnes et al., 2016; Valois et al., 2001, 2004a,b; Ye et al., 2014; Zullig et al., 2001).

The body of evidence supporting the relevance of youth SWB has prompted action at both national and international levels. International organizations such as the Organization for Economic Co-operation and Development (OECD), the World Health Organization (WHO), and the United Nations (UN) have developed dedicated policies and initiatives to monitor and enhance young people's wellbeing (Casas, 2011; Ross et al., 2020).

Whether for research, monitoring, or evaluation of life satisfaction-related interventions, reliable and valid measurement tools are required to assess SWB among youths. Several research teams have addressed this need by developing self-report scales that capture children's and adolescents views on their own wellbeing (see Cho and Yu, 2020; Losada-Puente et al., 2020 for comprehensive reviews).

### Measuring subjective wellbeing in youth populations: the Brief Multidimensional Students' Life Satisfaction Scale (BMSLSS) and its Chilean adaptation

Researchers have developed various instruments for measuring SWB in youth populations, which differ in their conceptual approach. Context-free or global measures such as the *Students' Life Satisfaction Scale* (SLSS, Huebner, 1991) assess overall life satisfaction without reference to specific domains. In contrast, domain-specific measures evaluate satisfaction across different life areas, including the *Personal Wellbeing Index-School Children* version (PWI-SC, Cummins and Lau, 2005), the *Multidimensional Students' Life Satisfaction Scale* (MSLSS, Huebner, 1994), the *Multidimensional Students' Life Satisfaction for adolescents* (Gilligan and Huebner, 2002), and the *Brief Multidimensional Students' Life Satisfaction Scale* (BMSLSS, Seligson et al., 2003).

In particular, the BMSLSS is quite popular. Initially it was developed as a concise alternative to the MSLSS to address time and resource constraints in large-scale surveys. Following the same theoretical model as the MSLSS but using only five items instead of forty, the BMSLSS assesses satisfaction with five specific life domains: (1) family life, (2) friendships, (3) school experience, (4) oneself, and (5) living environment. Each domain is represented by a single item, creating a brief yet comprehensive measure of children's and adolescents' life satisfaction across key areas. The original version employed a 7-point ordinal scale ranging from “*terrible*” to “*delighted*” for each item.

After its release by (Seligson et al. 2003), the BMSLSS was further validated in the USA with children and adolescents aged 8 to 18 (Huebner et al., 2004, 2006a,b, 2011). Subsequently, the scale has been used in many studies and adapted to different countries, exhibiting good psychometric properties such as item-test correlations, internal consistency reliability, test-retest reliability, and unidimensionality (Cho and Yu, 2020; Losada-Puente et al., 2020). Cross-cultural psychometric analysis reaches the same conclusions. For instance, (Abubakar et al. 2016) found that the scale shows good psychometric properties and a reasonable level of invariance across 23 countries. Related domain-based measures, such as the Children's Worlds Domain-Based Subjective Wellbeing Scale—which incorporates adapted BMSLSS items with a 0–10 response format—also demonstrate good psychometric properties across 35 countries (Casas and González-Carrasco, 2021).

Building on the international validation efforts, (Alfaro et al. 2015) validated a Spanish translation of the BMSLSS in Chile with a sample of Chilean students between 10 and 12 years old. Their study reported adequate reliability (alpha = 0.70) and a reasonable fit to a unidimensional confirmatory factor model. Notably, (Alfaro et al. 2015) modified the response format from the original 7-point scale to a 0–10 scale, and changed the anchors from “*terrible*”/“*delighted*” to “*totally dissatisfied*”/“*totally satisfied*”. These modifications were intended to increase the scale's sensitivity and adapt it to the Chilean context. More recently, the Chilean Ministry of of Social Development and Family, through its Early Childhood Longitudinal Survey (ELPI, 2017–2018), made another adaptation of the BMSLSS, in which they maintained the original 7-point response scale but modified the anchors to “*not satisfied at all*”/“*very satisfied*” (ELPI, 2017–2018). The impact of these response format modifications on item performance remains unexamined, largely due to methodological limitations.

This points to a broader unresolved issue in the psychometric analysis of child and adolescent SWB scales developed within the psychological and social sciences research traditions. While measures like the BMSLSS are widely used and validated, their psychometric properties have been studied primarily through exploratory or confirmatory factor analysis and Classical Test Theory (CTT) methods, such as test-retest reliability, internal consistency coefficients, and item-total correlations (Clark and Watson, 2019). This approach, though not inherently problematic, focuses on the reliability and validity of a composite score (i.e., the aggregate sum or average of its items) and may not provide optimal tools for detecting problems with item response alternatives. This limitation is particularly critical when using short scales like the BMSLSS, where all available information (i.e., all response patterns) is valuable. By prioritizing the covariance among items, classical methods may fail to fully utilize information from respondents' response patterns (Clark and Watson, 2019). Furthermore, most studies on SWB within the social sciences rely on small, non-random samples, which limit the external validity and generalizability of their findings.

### The advantages of modern psychometric techniques for analyzing subjective wellbeing scales

In contrast to CTT limitations, Item Response Theory (IRT, Van der Linden, 2018) offers distinct advantages for analyzing SWB measures in children and adolescents (Hays et al., 2021). IRT models offer a more sophisticated approach by estimating the relationship between an individual's response to an item and their level of the underlying latent trait. This allows for a more precise assessment of item properties and overall scale performance, providing valuable information that can be used to refine and optimize the measurement tool (Van der Linden, 2018).

The application of IRT methodology has proven fruitful in validating and improving several scales in the field, such as the KIDSCREEN (Erhart et al., 2010; Ravens-Sieberer et al., 2014), the KIDS-CAT (Devine et al., 2015), and the eudaimonic wellbeing scales of the PROMIS project (Forrest et al., 2019). These studies demonstrate the value of IRT in assessing the psychometric properties of child and adolescent SWB measures, enabling researchers to identify and address issues with item functioning, response categories, and scale dimensionality. The growing popularity of IRT models for evaluating self-report measures in recent years (Hays et al., 2021) underscores the importance of applying this advanced psychometric approach to the study of SWB in young populations.

The advantages of IRT models over classical psychometric methods have been well documented (Clark and Watson, 2019; Thomas, 2019). Four characteristics of IRT are particularly relevant for measuring SWB with brief scales in both adult and youth populations. First, IRT models estimate the probability of selecting each item response alternative as a function of the item properties and the latent trait level of the respondent. This approach allows for a detailed analysis of the discrimination power of each response option, providing information unavailable through classical methods. Second, IRT provides an optimal scoring rule for each answer option, weighting responses according to their discriminative power. This feature enhances the reliability of the results and maximizes the information extracted from the available data, which is especially critical when working with short scales like the BMSLSS. Third, IRT enables the estimation of reliability and measurement error conditional on different levels of the latent trait. Classical approaches provide only single reliability coefficients and standard errors that average across all trait levels, obscuring how measurement precision varies along the latent continuum (Kolen et al., 1992). Through conditional standard errors of measurement (CSEM), IRT identifies where along the BMSLSS scale the instrument provides its most precise measurements. This information proves particularly valuable when evaluating interventions, as it allows researchers to determine whether the scale can reliably detect changes for the specific population being targeted.

Another critical advantage of IRT methodology is that it provides a robust framework for testing differential item functioning (DIF), a form of measurement non-invariance that occurs when items perform differently across subgroups defined by a third variable, such as gender or age (Millsap, 2012; Osterlind and Everson, 2009). DIF can pose a serious threat to scale validity, as it suggests that item responses are influenced by factors other than the latent trait being measured.

For instance, evidence of gender DIF would indicate that boys and girls with the same level of SWB have different probabilities of selecting a particular response option solely due to gender. Notice that current evidence about gender differences in SWB among children and adolescents shows an inconsistent pattern. A recent meta-analysis (Chen et al., 2020) found no differences between boys and girls in overall life satisfaction but revealed gender variations in domain-specific satisfaction: girls typically report higher satisfaction with school and friends, while boys report higher satisfaction with themselves. This raises questions about how these domain-specific differences might affect the measurement properties of scales like the BMSLSS, which aggregate satisfaction across life domains. DIF analysis becomes crucial for addressing such measurement concerns.

Age-related DIF presents another critical consideration. If a scale lacks age invariance, response probabilities would depend not only on SWB levels but also on respondent age. This issue is particularly relevant given the consistent finding that SWB decreases during adolescence, specifically between ages 10 and 16 (Casas and González-Carrasco, 2020, 2022; González-Carrasco et al., 2017b; Handa et al., 2023; Orben et al., 2022). Longitudinal studies indicate this decline begins around ages 11–12 and appears more pronounced when measured with domain-based scales like the BMSLSS (González-Carrasco and Casas, 2024; González-Carrasco et al., 2017a). If this effect manifests uniformly across items, the scale may maintain invariance. However, if certain items show differential sensitivity to this decline, the scale could exhibit non-invariance, compromising score comparability across age groups. Formal testing of age-related DIF is essential to determine if BMSLSS responses are comparable across different ages.

To address these issues and ensure BMSLSS comparability across gender and age subgroups, a thorough examination of DIF is essential. While traditional approaches to testing measurement invariance, such as multi-group confirmatory factor analysis (Hashim and Areepattamannil, 2017; Millsap, 2012), can be applied within the IRT framework, Multiple Indicators Multiple Causes (MIMIC) models have emerged as an increasingly popular alternative (Finch, 2005; Woods and Grimm, 2011). MIMIC models examine DIF by modeling two potential causal pathways through which variables like gender or age might affect measurement (Montoya and Jeon, 2020): their effect on the latent trait (e.g., a direct influence of age on life satisfaction) and their effect on specific item responses after controlling for the latent trait level (DIF). This approach can be implemented either as an IRT model fitted directly to the data or as a confirmatory factor analysis using polychoric correlations (Muthén et al., 1991; MacIntosh and Hashim, 2003). The ability to separate true effects on the latent trait from artifactual effects on item responses, combined with their flexibility in model specification, makes MIMIC models particularly suitable for examining DIF in measures like the BMSLSS.

### The present study

In summary, despite the advantages of IRT methodology, its application to children's and adolescents' SWB scales remains limited, particularly for widely used instruments like the BMSLSS. Notably, we found only one study claiming to use IRT for psychometric analysis of the BMSLSS, but this study actually applied factor analysis for ordinal items (Pittman et al., 2021). In the present research, we examine the psychometric properties of the BMSLSS using data from a large, representative sample of Chilean early adolescents who participated in the third round of the *Longitudinal Early Childhood Survey* (*Encuesta Longitudinal de Primera Infancia*, ELPI). We examine item response functions, item and total precision, and investigate differential item functioning related to both gender and age in this widely used scale. Through this analysis, we aim to contribute to the understanding of the BMSLSS's measurement properties and provide empirically based recommendations for its use in research and assessment with early adolescent populations.

## Method

### Participants and procedure

We analyzed data from the third wave of the Chilean Early Childhood Longitudinal Survey (ELPI, 2017–2018), a nationally representative study conducted by the Chilean Ministry of Social Development and Family to examine developmental outcomes and sociodemographic characteristics of Chilean children and their families. Data collection involved two household visits where trained undergraduate psychology students, serving as research assistants, administered a battery of instruments assessing cognitive, social, and emotional variables. Data quality was ensured through comprehensive training of research assistants and rigorous protocols for data capture, processing, and storage, as detailed in the study's technical manuals (ELPI, 2018).

For participants aged 10–12 (classified as early adolescents; see Lansford and Banati, 2018), the protocol included a self-report questionnaire containing the BMSLSS and other scales. The ELPI study received approval from multiple ethics committees. A dual consent procedure was employed: primary caregivers (parents/mothers or legal guardians) provided written informed consent, and additionally, the children who completed the self-report questionnaire provided written informed assent (ELPI, 2018).

Participants were 5,619 early adolescents (49.2% girls, 50.8% boys) aged 10 (46.13%), 11 (44.99%), and 12 years (8.88%). They attended public (48.83%), voucher-subsidized (46.98%), and private schools (4.19%), distributed across second (0.43%), third (3.17%), fourth (20.14%), fifth (41.82%), sixth (27.27%), seventh (7.12%), and eighth (0.05%) grades of primary education, which in Chile is compulsory and spans eight grades for students aged 6–14.

### Measures

The *Brief Multidimensional Students' Life Satisfaction Scale* (BMSLSS; Seligson et al., 2003) is a five-item self-report instrument that assesses satisfaction across five life domains: (1) family life, (2) friendships, (3) school experience, (4) oneself, and (5) living environment. While a Chilean adaptation exists (Alfaro et al., 2015) based on the Spanish version (Casas et al., 2012), the ELPI research team developed their own translation of the original scale from (Seligson et al. 2003). This ELPI version maintains the original 7-point ordinal response format but modifies the response anchors. Instead of using the original labels ranging from 1= “*terrible*” to 7 = “*delighted*”, the ELPI version employs anchors from 1 = “*not satisfied at all*” to 7 = “*very satisfied*” (in Spanish: “*Nada Satisfecho*” to “*Muy satisfecho*”). Despite an exhaustive review of ELPI documentation, we found no explicit rationale for this modification of the response anchors from the original (Seligson et al. 2003) version. Notice that the ELPI response format also differs from the previous Chilean adaptation by (Alfaro et al. 2015), which used an 11-point scale ranging from 0 = “*totally dissatisfied*” to 10 = “*totally satisfied*” (in Spanish: “*Totalmente Descontento*” to “*Totalmente Contento*”).

### Analytical strategy: Samejima's Graded Response Model and MIMIC model

IRT comprises many different models. Given the presumed unidimensionality of BMSLSS and its seven ordinal response options, we selected the unidimensional version of the Graded Response Model (GRM; Samejima, 1997) for our analyses. GRM is typically the model of choice for ordinal items due to its superior fit properties (Ostini et al., 2014).

The fundamental aim of IRT is to estimate the probability of selecting specific item responses as a function of both item properties and the respondent's latent trait level. In the context of BMSLSS, this means modeling the probability of selecting each response option (from “*not satisfied at all*” to “*very satisfied*”) conditional on the respondent's underlying level of the construct being measured by the scale as a whole. The GRM addresses this by modeling the probability of responding in or above each response category according to the equation:


$$\begin{array}{l} P\left(x\geq k|\theta \right)=\frac{1}{1+\text{exp}\left[-a_{j}\left(\theta -b_{jk}\;\right)\right]}. \end{array}$$

This equation indicates that the probability of responding at or above category *k* (e.g., “*not satisfied at all*”) in item *j*, depends on two types of item parameters (*a* and *b*), conditional on the latent trait (θ) being measured.

The latent trait (θ) represents the underlying continuous variable (life satisfaction in this case) that the items measure. During calibration θ it is typically constrained to a mean of zero and variance of 1 to fix the scale of the latent variable, allowing its interpretation as a z-score. The *discrimination* parameter (*a*) describes the strength of the relationship between each item and the latent trait, with higher values indicating better discrimination between respondents at different levels of the construct. This parameter is mathematically related to factor loadings (λ) through the equation (Takane and De Leeuw, 1987; Wirth and Edwards, 2007):


$$\begin{array}{l} \lambda_{j}=\frac{a_{j}/D}{\sqrt{1+{\left(a_{j}/D\right)}^{2}}} \end{array}$$

where *D* is a scaling constant of 1.7, that accounts for the different distributional assumptions between GRM (logistic) and factor analysis (normal).

The *category thresholds* or *category location* parameters (*b*_*jk*_), indicate the point on the latent trait scale where respondents have a *p* = 0.5 probability of selecting a given response option or higher. For an item with *k* response options, the GRM estimates *k*−*1 threshold* parameters. Given the seven response options in BMSLSS, we estimate six threshold parameters per item. These parameters share the latent trait's metric and represent the satisfaction level needed to select successive response options with probability greater than 0.5. For example, *b*_1_ describes the probability of selecting response options 2, 3, 4, 5, 6, or 7; *b*_2_ the probability of choosing response options 3, 4, 5, 6, or 7; etcetera. Values of category *thresholds* are estimated in the same metric as the latent trait score (θ). The probability of selecting specific categories is obtained by subtracting adjacent category probabilities, typically visualized through *Category Characteristic Curves* (CCC).

It is worth noting that some authors prefer the *intercept* parameter notation instead of the more conventional *threshold* parameter nomenclature. For each response option, the intercept parameter, *c*, is defined as *c* = –*a*_*j*_*b*_*jk*_ and can be interpreted as the log-odds of responding at or above the response category when the latent trait (θ) is zero, that is, equal to the mean (because θ has a standardized metric similar to z-scores).

**Figure 1:**
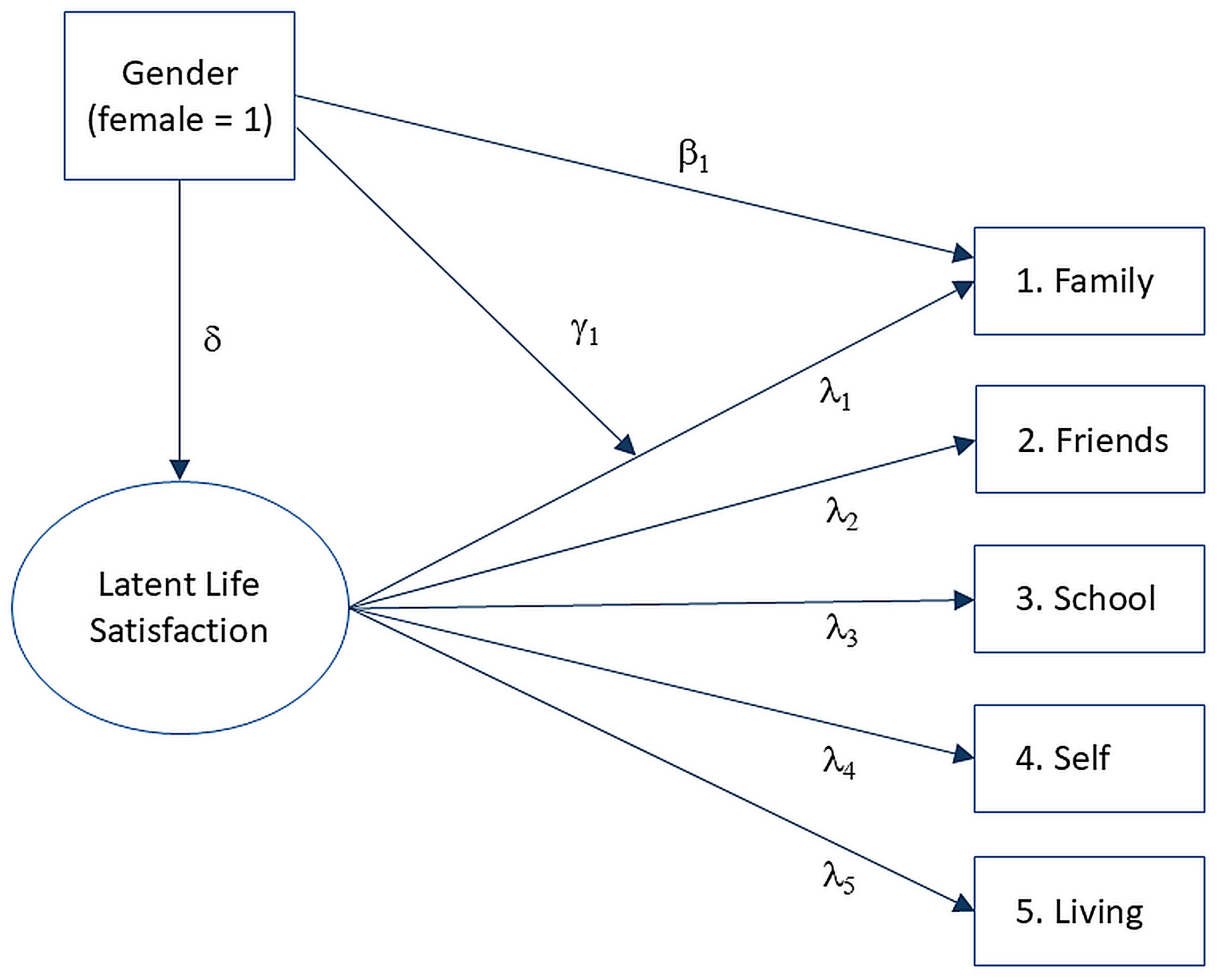
Simplified path diagram of the MIMIC model for estimating gender uniform and non-uniform DIF effects on item 1. Note For clarity, item measurement errors are omitted. δ = effect of gender (being female) on the latent construct; β = direct effect (uniform DIF) of gender on item 1, holding constant its effect on the latent construct; γ = moderation effect (non-uniform DIF) of gender on item 1 discrimination (or loading, λ).

To examine measurement invariance, we extended this GRM framework using a Multiple Indicators Multiple Causes (MIMIC) model. Following (Montoya and Jeon 2020), our MIMIC model combines two components: a measurement model relating items to the latent trait (the GRM described above), and a structural model incorporating the effects of covariates (gender and age) on both the latent trait and individual items. Figure 1 presents a simplified path diagram of this model for gender.

The analysis proceeds in three steps. First, we estimate the structural path (δ) representing gender's effect on the latent life satisfaction variable. Second, we estimate the direct path (β) from gender to each item while controlling for gender's effect on latent life satisfaction. This direct effect (β) represents what DIF literature calls *uniform DIF*: when gender influences the probability of selecting higher or lower response options beyond the true level of latent life satisfaction. Third, we estimate the interaction path (γ) that represents how gender moderates the relationship between the latent trait and item responses. This interaction captures *non-uniform DIF*: the extent to which gender affects an item's discrimination parameter after controlling for both the effect of gender on latent life satisfaction (δ) and its direct effect on item responses (β). Non-uniform DIF indicates that the item's ability to differentiate between levels of life satisfaction varies by gender.

Given the multiple significance tests involved in DIF analyses and our large sample size, we adopted a more conservative threshold (α = 0.01) to reduce Type I error rates while maintaining adequate power. This approach aligns with recommendations for DIF analysis in large-scale assessments (e.g., Stark et al., 2006) where traditional significance levels may lead to the detection of trivial effects.

All analyses were conducted using multiple software packages: Stata 18 (StataCorp, 2023) was used for data processing and cleaning, descriptive analyses, and generation of all figures and tables presented in this manuscript. R version 4.2.2 (R Core Team, 2022) with the mirt package (Chalmers, 2012) was employed for Item Response Theory (IRT) analyses including the Graded Response Model. Mplus version 7.4 (Muthén, L. K., and Muthén, 1998–2024) was used for the Multiple Indicators Multiple Causes (MIMIC) models.

## Results

### Descriptive statistics and classical test theory analysis

Prior to conducting the analyses, we examined missing data patterns across the BMSLSS items. Only 6 participants (0.1%) had missing values on any BMSLSS item. We employed listwise deletion, resulting in a final analytical sample of 5,613 participants with complete BMSLSS data.

Table 1 presents descriptive statistics for the BMSLSS items. Response patterns showed a clear preference for the higher end of the scale (options 5–7), with particularly high endorsement of the maximum satisfaction level (option 7) across all life domains. Satisfaction with family life (item 1) exhibited the most positive evaluations, with a mean of 6.49 (SD = 1.05) and 70.92% of respondents selecting the highest response option (“*very satisfied*”). Satisfaction with oneself (item 4) and satisfaction with friend (item 2) also showed high ratings, with means of 6.34 (SD = 1.22) and 6.28 (SD = 1.18) respectively, and more than 59% of respondents choosing the maximum satisfaction level in both domains. While satisfaction with school (item 3, *M* = 6.17, SD = 1.20) and satisfaction with the living environment (item 5, *M* = 5.91, SD = 1.53) maintained positive evaluations in general, they showed slightly lower means and more dispersed distributions compared to other satisfaction with life domains, with approximately 50% of respondents selecting the highest response option. The overall scale mean, calculated as the average across all items, was *M* = 6.24 (SD = 0.86).

**Table 1:** Means, standard deviations and endorsement frequency (%) for each alternative of BMSLSS items.

**Item**	**Mean**	**SD**	**Endorsement frequency (%)**						
			**RO1**	**RO2**	**RO3**	**RO4**	**RO5**	**RO6**	**RO7**
1. Family	6.49	1.05	1.30	0.49	0.77	2.30	6.89	17.33	70.92
2. Friends	6.28	1.18	1.66	0.77	1.20	3.12	9.82	24.19	59.24
3. School	6.17	1.20	1.67	0.86	1.32	4.24	11.22	28.54	52.15
4. Self	6.34	1.22	2.12	0.71	1.21	2.94	8.08	20.27	64.67
5. Living environment	5.91	1.53	3.92	1.55	2.99	5.45	13.63	21.95	50.51

*N* = 5,613. RO, Response option.

As shown in Table 2, inter-item correlations averaged 0.37, demonstrating magnitudes consistent with those reported by (Alfaro et al. 2015) in their adaptation. The table also presented corrected item-total correlations, which assess items' discriminative capacity. All values exceed *r*_rest_ = 0.30, indicating strong relationships between individual items and the overall scale score.

**Table 2:** Classical item analysis for the BMSLSS: Item-rest correlations, factor loadings (λ) and inter-item correlations.

**Item**	***r*_rest_**	**λ**	**Inter-item correlations**				
			**Item 1**	**Item 2**	**Item 3**	**Item 4**	**Item 5**
1. Family	0.57	0.75	1.0				
2. Friends	0.52	0.73	0.43	1.0			
3. School	0.49	0.71	0.39	0.42	1.0		
4. Self	0.55	0.83	0.51	0.39	0.39	1.0	
5. Living environment	0.39	0.70	0.32	0.29	0.26	0.32	1.0

All correlations are significant at *p* < 0.001.

Dimensionality analyses supported a unidimensional structure. Principal component analysis revealed a clear first factor, with the ratio of first to second eigenvalues (2.50/0.77 = 3.25) suggesting dominant unidimensionality. A maximum likelihood confirmatory factor analysis of a unidimensional model corroborated this finding, showing good model fit (CFI = 0.985; TLI = 0.971; RMSEA = 0.055, 90% CI [0.045, 0.065]). Factor loadings, presented in Table 2′s third column, range from 0.70 for living environment satisfaction (item 5) to 0.83 for satisfaction with oneself (item 4).

Finally, internal consistency analyses yielded comparable results across three reliability coefficients: Cronbach's alpha (α = 0.73), McDonald's omega (ω = 0.73), and the Greatest Lower Bound of reliability (GLB = 0.75). These values indicate acceptable reliability levels and align with those reported in the previous Chilean adaptation of the BMSLSS (Alfaro et al., 2015).

These preliminary analyses suggested that the ELPI version of BMSLSS exhibits sound psychometric properties by conventional criteria (Clark and Watson, 2019), performing similarly to previous Chilean adaptation (Alfaro et al., 2015).

### Item Response Theory analysis

After establishing the basic psychometric properties of the BMSLSS, we proceeded with the IRT analysis using Samejima's Graded Response Model, as specified in the Method section.

Model fit evaluation represents a crucial step in IRT analysis, as it determines whether the mathematical model adequately captures the response patterns in the data. We assessed global model fit using M2 statistics and RMSEA (Maydeu-Olivares and Joe, 2006; Maydeu-Olivares, 2015). The results (M2 = 43.46, d*f* = 5, *p* < 0.001; RMSEA = 0.03) indicate that the graded response model provides a good representation of the response patterns in our data. We also examined model fit at the item level using the S–*X*^2^ statistic (Orlando and Thissen, 2003) and its associated RMSEA, as shown in Table 3. The RMSEA values for all items indicate excellent fit, suggesting that the GRM adequately represents the response patterns for each BMSLSS item.

**Table 3:** Item fit statistics for the Graded Response Model.

**Item**	**S–*X*^2^**	**d*f***	**RMSEA**
1. Family	172.06	84	0.014
2. Friends	197.90	88	0.015
3. School	207.21	88	0.016
4. Self	186.84	83	0.015
5. Living environment	150.35	87	0.011

After confirming the model's adequate fit to the data, we examined the item parameters estimated by the GRM, presented in Table 4. *Discrimination parameters* (*a*) reveal that satisfaction with family life (*a* = 2.32) most effectively distinguished between respondents with low and high satisfaction levels, followed by satisfaction with oneself (*a* = 1.99). This pattern is also reflected in their IRT-derived factor *loadings* (λ = 0.81 and λ = 0.76, respectively). In contrast, satisfaction with living environment showed substantially lower discrimination (*a* = 1.05, λ = 0.53), indicating it contributed least to the measurement of the common construct underlying all five items.

**Table 4:** IRT analysis for the BMSLSS: item loadings (λ), items slopes (*a*) category thresholds (*b*_*k*_) and intercepts (*c*_*k*_).

**Item**	**λ**	***a***	**Category thresholds**						**Intercepts**					
			***b**_1_*	***b**_2_*	***b**_3_*	***b**_4_*	***b**_5_*	***b**_6_*	***c**_1_*	***c**_2_*	***c**_3_*	***c**_4_*	***c**_5_*	***c**_6_*
1. Family	0.81	2.32	−2.98	−2.77	−2.53	−2.09	−1.46	−0.67	6.89	6.41	5.85	4.84	3.37	1.55
2. Friends	0.68	1.58	−3.39	−3.09	−2.75	−2.22	−1.40	−0.33	5.35	4.87	4.34	3.51	2.20	0.52
3. School	0.66	1.50	−3.48	−3.14	−2.79	−2.14	−1.29	−0.08	5.21	4.71	4.18	3.21	1.94	0.12
4. Self	0.76	1.99	−2.84	−2.64	−2.37	−1.96	−1.34	−0.49	5.65	5.24	4.72	3.90	2.67	0.96
5. Living environment	0.53	1.05	−3.53	−3.17	−2.67	−2.07	−1.12	−0.03	3.71	3.33	2.81	2.17	1.17	0.03

The *threshold parameters* (*b*) provide particularly informative insights into the scale's functioning. Across all items, the highest thresholds (*b*_6_) were notably close to zero, ranging from −0.67 (family) to −0.03 (living environment), indicating that the probability of selecting the maximum response option (7 = “*very satisfied*”) increases only at near-average or above-average levels of life satisfaction. Additionally, the small distances among the lower thresholds (*b*_1_, *b*_2_, and *b*_3_) suggested limited discriminative power among the first response options. For instance, for the family satisfaction item, thresholds *b*_1_ through *b*_3_ cluster between −2.98 and −2.53, spanning less than half a standard deviation on the latent trait scale.

**Figure 2:**
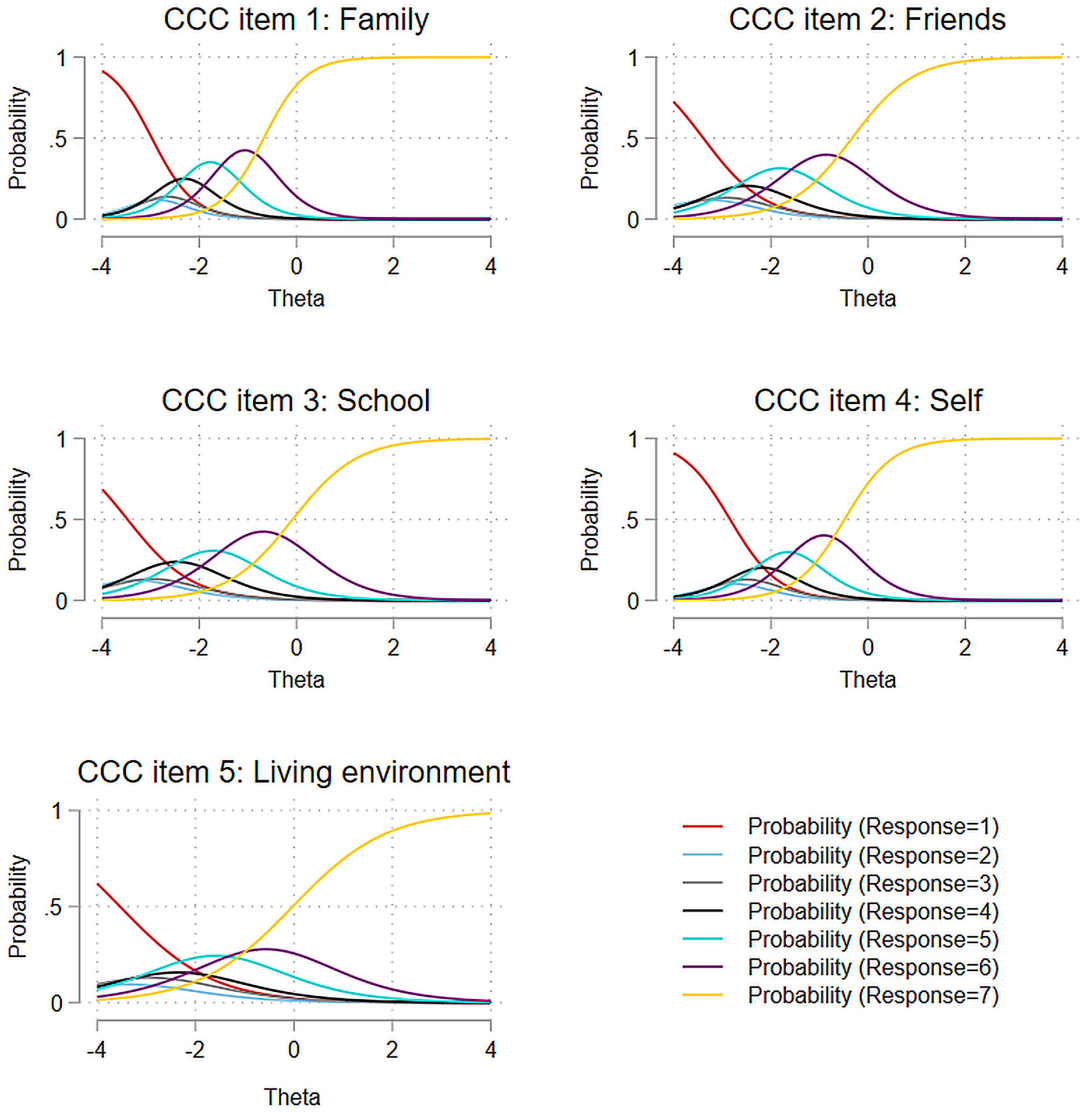
Category characteristic curves of BMSLSS items.

Visual inspection of the Category Characteristic Curves (CCC) in Figure 2 provides deeper insights into these patterns. The curves revealed that response options 1 through 4 lack clearly distinguishable regions of maximum probability along the latent trait continuum. Furthermore, the probability curves for response options 1 through 6 concentrated primarily in the negative region of the latent trait scale (θ < 0), indicating these options were most likely to be selected by respondents with lower levels of the underlying construct.

**Figure 3:**
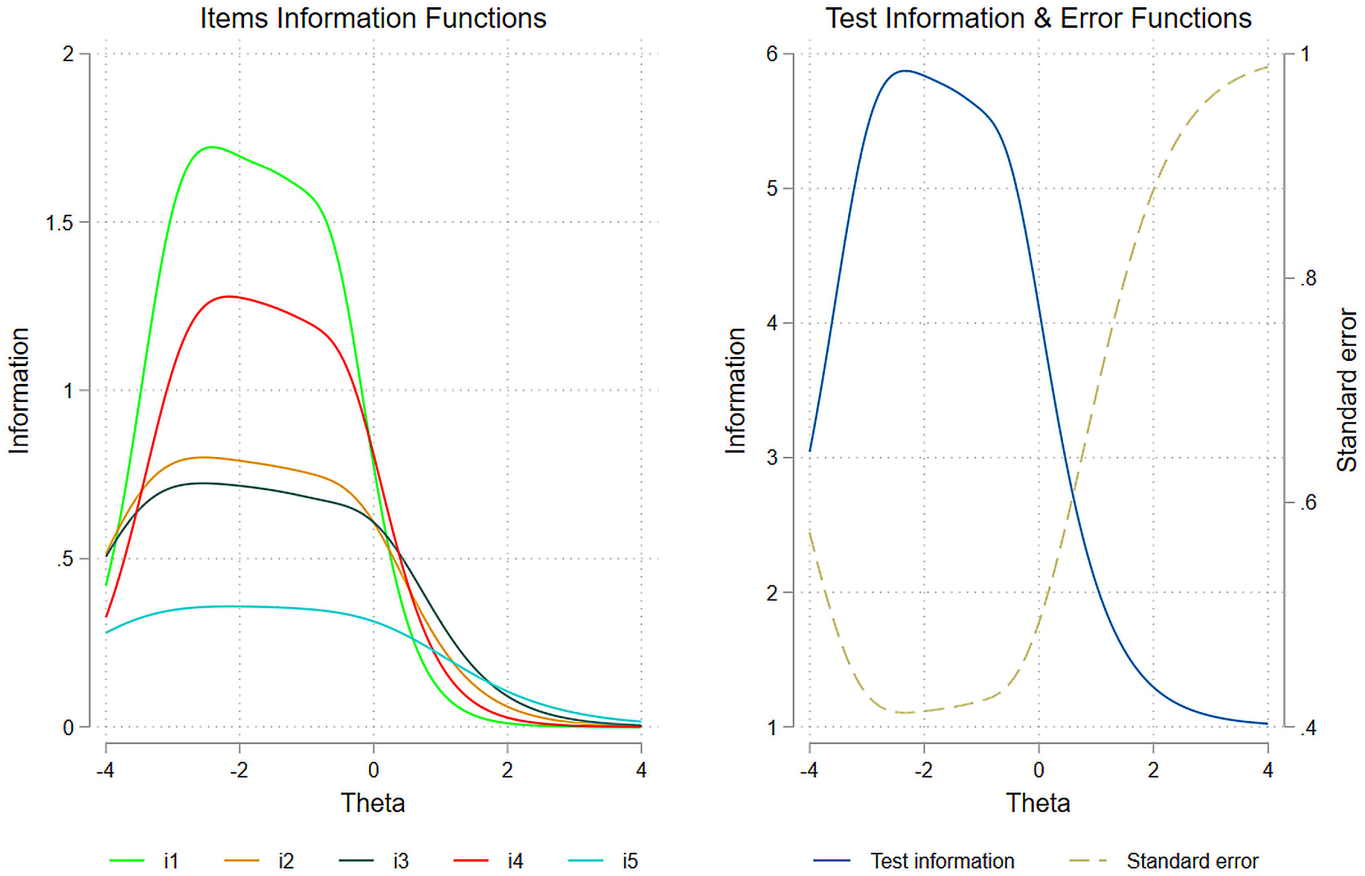
Items and test information functions and conditional standard error of measurement of the BMSLSS.

Figure 3 presents the item and test information functions, along with conditional standard errors of measurement. Item Information Functions (IIF) represent the IRT analog to reliability, with two key distinctions: they are estimated at the item level rather than the scale level, and they provide a continuous function of measurement precision across the latent trait continuum rather than a single index.

The left part of Figure 3 displays IIFs for each BMSLSS item. Consistent with the discrimination parameters reported in Table 4, satisfaction with family life (item 1) provided the most reliable information about the latent trait, while satisfaction with living environment (item 5) contributed the least. Notably, all IIFs showed leftward displacement relative to the latent construct (θ) distribution, indicating higher measurement precision for respondents with below-average life satisfaction levels. This pattern suggests that the BMSLSS items provide less reliable information for children and adolescents reporting higher levels of life satisfaction.

The right part of Figure 3 presents both the Test Information Function (TIF), which aggregates individual IIFs to represent overall scale reliability, and the Conditional Standard Error of Measurement (CSEM). The TIF confirms that scale precision decreased at higher levels of life satisfaction. The CSEM, calculated as the reciprocal of the square root of the TIF, provides a more familiar metric of measurement precision. As evident in the figure, measurement error increases substantially for respondents with above-average life satisfaction levels.

These IRT findings provide a more nuanced understanding of the scale's reliability than traditional CTT analyses. While the overall reliability coefficients (α = 0.73, ω = 0.73, GLB = 0.75) suggested acceptable measurement precision, the IRT analysis reveals that precision varies substantially across different levels of the latent trait. Specifically, the BMSLSS provides more reliable measurements for respondents with low to moderate life satisfaction but becomes less precise at higher levels. This pattern, not detectable through classical reliability indices, has important implications for the scale's use in research and assessment contexts, particularly when evaluating interventions targeting children and adolescents with high levels of life satisfaction.

### Differential item functioning across gender and age

We first examined gender differences in BMSLSS items. Results are presented in Table 5. Zero-order correlations between gender (coded as female = 1, male = 0) and item responses were negligible (*r* ≤ |0.03|), suggesting minimal raw gender differences in domain-specific life satisfaction ratings. However, the MIMIC analysis revealed more complex patterns.

**Table 5:** Item Differential Functioning (DIF) by gender: MIMIC-based estimation of uniform (β) and non-uniform (γ) DIF effects.

**Item**	***r***	***p***	**Uniform DIF effect**			**Non-uniform DIF effect**		
			β	***S.E***.	***p***	γ	***S.E***.	***p***
1. Family	−0.02	0.16	0.11	0.11	0.35	0.31	0.13	0.02
2. Friends	0.00	0.79	0.05	0.07	0.51	0.04	0.09	0.70
3. School	0.03	0.03	**0.33**	0.07	**< 0.001**	**0.24**	0.08	**< 0.001**
4. Self	−0.03	0.04	−0.01	0.09	0.96	0.26	0.11	0.02
5. Living environment	−0.03	0.06	−0.08	0.06	0.19	0.15	0.07	0.04

Significant effects with alpha = 0.01 in bold. Female = 1; positive DIF values imply that the effect favors girls over boys. r = zero-order correlation between item and gender, *p* = significance. β = gender effect on the odds of choosing a higher alternative on the item, holding constant latent life satisfaction. γ = gender effect on item discrimination, holding constant latent life satisfaction.

Only satisfaction with school (item 3) displayed significant uniform DIF (β = 0.33, SE = 0.07, *p* < 0.001), indicating that, controlling for the latent construct level, girls were more likely than boys to select higher response options when rating their school satisfaction. This item also showed significant non-uniform DIF (γ = 0.24, SE = 0.08, *p* < 0.001), suggesting that its discriminating power varies by gender, with stronger discrimination among girls. In other words, school satisfaction contributes more strongly to overall life satisfaction for girls than for boys.

**Figure 4:**
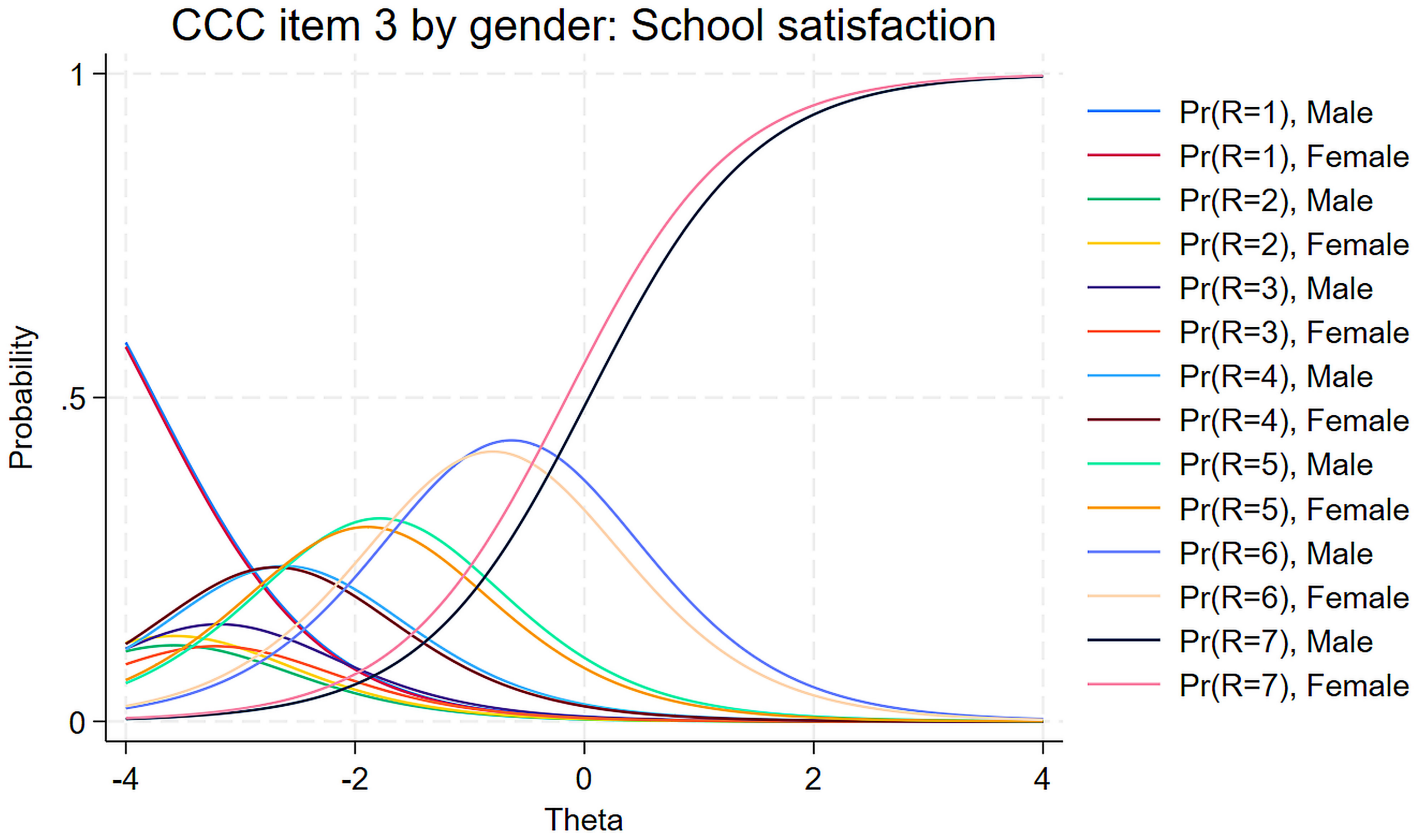
Comparison of category characteristic curves of item 3, school satisfaction, for girls and boys.

Figure 4 illustrates these gender differences in school satisfaction through Category Characteristic Curves. The curves for the highest response category (7 = “*very satisfied*”) showed clear separation between males and females across most of the latent construct range, with the female curve (pink) consistently above the male curve (black). This visualization illustrates the uniform DIF effect: at any given level of the latent construct, girls are more likely than boys to select the highest satisfaction rating for school. Additionally, the steeper slopes of the curves for females, particularly visible in the middle range of the latent construct (Θ between −1 and 1), demonstrated the non-uniform DIF effect: the relationship between school satisfaction ratings and the latent construct is stronger for girls than for boys.

As shown in Table 5, three additional items showed weaker evidence of non-uniform DIF across gender: satisfaction with family (γ = 0.31, SE = 0.13, *p* = 0.02), satisfaction with oneself (γ = 0.26, SE = 0.11, *p* = 0.02), and satisfaction with living environment (γ = 0.15, SE = 0.07, *p* = 0.04). However, as these *p*-values exceed our predetermined significance threshold of α = 0.01, established following (Stark et al. 2006) as described in the Method section, and therefore they did not constitute conclusive evidence of differential item functioning.

Next, we examined age-related differential item functioning for the BMSLSS items. Results are presented in Table 6. Zero-order correlations between age and item responses were negligible (all *r* ≤ |0.02|), indicating minimal raw age differences in domain satisfaction ratings.

**Table 6:** Item Differential Functioning (DIF) by age: MIMIC-based estimation of uniform (β) and non-uniform (γ) DIF effects.

**Item**	***r***	***p***	**Uniform DIF effect**			**Non-uniform DIF effect**		
			β	***S.E***.	***p***	γ	***S.E***.	***p***
1. Family	−0.01	0.51	0.01	0.02	0.77	0.00	0.01	0.89
2. Friends	−0.01	0.40	0.00	0.01	0.91	−0.01	0.01	0.15
3. School	0.00	0.93	−0.02	0.01	0.02	−0.01	0.01	0.09
4. Self	−0.02	0.20	−0.07	0.03	0.02	−0.02	0.02	0.38
5. Living environment	0.01	0.45	–**0.02**	0.01	**0.01**	–**0.02**	0.01	**< 0.001**

Significant effects with alpha = 0.01 in bold. Age was measured in months; positive DIF values imply that the effect increases with age. r = zero-order correlation between item and gender, *p* = significance. β = age effect on the probability of choosing a higher alternative on the item, holding constant latent life satisfaction. γ = age effect on item discrimination, holding constant latent life satisfaction.

The MIMIC analysis revealed that only satisfaction with living environment (item 5) exhibited significant DIF effects. This item showed both uniform DIF (β = −0.02, SE = 0.01, *p* = 0.01) and non-uniform DIF (γ = −0.02, SE = 0.01, *p* < 0.001). The negative uniform DIF coefficient indicates that, at equal levels of the latent construct, older respondents were less likely to select higher response options when rating their satisfaction with living environment. In practical terms, this means that even when two respondents—one younger and one older—have the same underlying level on the latent construct, the older respondent tends to report lower satisfaction with their living environment. The negative non-uniform DIF coefficient suggests that the relationship between living environment satisfaction and the latent construct weakens slightly with age, indicating that this domain contributes less to the latent construct as adolescents get older.

**Figure 5:**
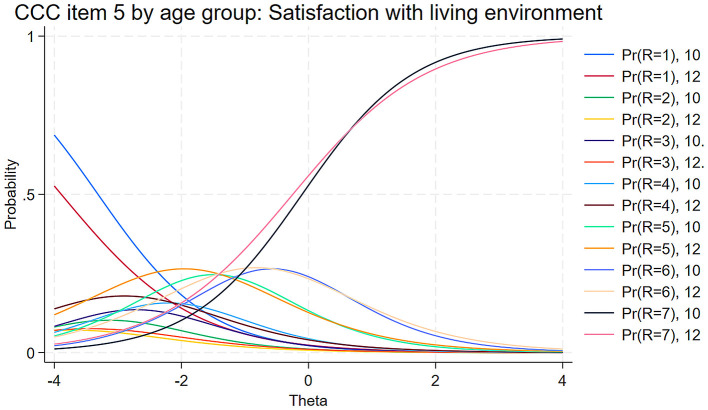
Comparison of category characteristic curves of item 5, satisfaction with the living environment, for 10 and 12 years olds.

Figure 5 provides a visual representation of these age-related differences in living environment satisfaction through category characteristic curves, comparing responses at ages 10 and 12. The uniform DIF effect is visible in the rightward shift of the curves for 12-year-olds, particularly noticeable in the highest response category (7 = “*very satisfied*”). This shift indicates that older respondents require higher levels of the latent construct to endorse the maximum satisfaction with their living environment. Additionally, the flatter curves for 12-year-olds, especially in the middle range of the latent trait (θ between −1 and 1), illustrate the non-uniform DIF effect: the relationship between living environment ratings and the latent construct becomes less pronounced with age.

Finally, as indicated in Table 6, although satisfaction with school (β = −0.02, SE = 0.01, *p* = 0.02) and satisfaction with oneself (β = −0.07, SE = 0.03, *p* = 0.02) showed some evidence of uniform DIF across age, these *p*-values exceed our predetermined significance threshold of 0.01. Similar to our gender DIF findings, these results suggest potential measurement differences that, while not meeting our strict significance criterion, indicate areas that merit attention in future research.

## Discussion

This study represents the first application of Item Response Theory (IRT), specifically the Graded Response Model (GRM), to evaluate the psychometric properties of the Brief Multidimensional Students' Life Satisfaction Scale (BMSLSS). While conventional psychometric analyses, such as item-test correlations, internal consistency, and factor analysis, indicated good overall performance of the BMSLSS, the IRT approach revealed nuances in the scale's functioning that were not detected by classical methods. The GRM allowed for a more detailed examination of the response categories and provided insights into areas for potential improvement in this adaptation of the BMSLSS for Chilean early adolescents.

### Main findings: psychometric limitations of the BMSLSS

The main limitation of the ELPI version of the BMSLSS, as revealed by our IRT analysis, is the psychometric quality of the response alternatives. The GRM showed that the lower response categories, particularly “*not satisfied at all*”, do not effectively discriminate among respondents and are largely redundant. Conversely, at the high end of the life satisfaction spectrum, the response option “*very satisfied*” appears insufficient to capture nuances in respondents' life satisfaction levels.

From a substantive perspective, one possible interpretation of these patterns relates to the optimism bias (Weinstein, 1980) commonly documented in children and adolescents, whereby young people tend to evaluate their life circumstances more positively than adults (Tetzner and Becker, 2025). This developmental tendency toward positive self-evaluation could explain both the underutilization of lower response categories and the concentration of responses at the upper end of the scale.

### Factors contributing to response scale limitations

From a psychometric perspective, however, these response patterns create measurement challenges that warrant examination of the scale's design. Several factors may contribute to these psychometric issues. It is important to note that, as we are working with an adapted version of the BMSLSS, we cannot unequivocally attribute these findings to inherent characteristics of the original scale, specificities of the ELPI adaptation, or cultural factors unique to the Chilean sample. Nevertheless, a close examination of the modifications made in the ELPI version suggests potential explanations for the observed limitations in the response scale.

One key difference between the ELPI adaptation and previous versions of the BMSLSS is the number of response categories. The ELPI version employed a 7-point scale, whereas the extant Chilean adaptation by (Alfaro et al. 2015) used an 11-point scale, following the recommendations of (Casas et al. 2012). Drawing on the work of (Cummins and Gullone 2000), (Casas et al. 2012) argue that increasing the number of response alternatives does not compromise scale reliability, but rather enhances sensitivity and allows for better discrimination between respondents. Therefore, the use of only seven response options in the ELPI version may be insufficient to capture the full range of life satisfaction, particularly among highly satisfied respondents.

The labels used for the scale anchors in the ELPI adaptation may also contribute to the psychometric limitations observed. In the original BMSLSS (Seligson et al., 2003), the response alternatives ranged from “*terrible*” to “*delighted*”, while the Chilean adaptation by (Alfaro et al. 2015) used labels from “*totally unhappy*” to “*totally happy*”. These labels convey a higher degree of affective intensity compared to the ELPI version, which employed the anchors “*not satisfied at all*” and “*very satisfied*”. Research has shown that category labels can significantly influence item responses (Weng, 2004), and labels with low emotional intensity connotations may not adequately capture extreme positions on the subjective continuum of life satisfaction. Consequently, the label “*very satisfied*” in the ELPI version may not sufficiently discriminate among respondents who are highly satisfied with their lives.

Another noteworthy difference between the ELPI adaptation and previous versions of the BMSLSS is the use of a unipolar scale rather than a bipolar one. In the original BMSLSS as well as in previous adaptations, the response alternatives comprised antithetical adjectives, such as “*terrible*” vs. “*delighted*” and “*totally unhappy*” vs. “*totally happy*”. In contrast, the ELPI version's anchors, “*not satisfied at all*” and “*very satisfied*”, do not represent true antonyms. While the evidence is not conclusive, some studies suggest that responses to the same items can differ between bipolar and unipolar scales (Steinberg and Rogers, 2020). This difference in scale polarity may further contribute to the psychometric limitations observed in the ELPI adaptation. At the same time, unipolar scales may be more developmentally appropriate for children, as they require less cognitive complexity to understand and use effectively (Borgers et al., 2000; Mellor and Moore, 2014).

Finally, cultural factors specific to the Chilean context may influence how early adolescents interpret and respond to the BMSLSS items. In the Chilean school system, grades range from 1 to 7, with 4 being the minimum passing grade. This grading scale may create implicit connotations that affect how students perceive and use the response options on the BMSLSS. For example, students may be reluctant to select options 1–3, as these grades are typically associated with poor academic performance. This cultural norm may contribute to the redundancy of the lower response categories observed in the IRT analysis.

Summarizing, the psychometric limitations of the ELPI version of the BMSLSS, particularly the redundancy of lower response categories and the insufficient discrimination at the high end of the life satisfaction spectrum, may be attributed to a combination of factors. These include the reduced number of response options compared to previous adaptations, the use of less intense labels for scale anchors, the unipolar nature of the response scale, and cultural norms specific to the Chilean educational context. Further research is needed to disentangle the relative contributions of these factors and to develop an optimal response scale for measuring life satisfaction among Chilean early adolescents.

### Practical implications and recommendations

Regardless of the underlying causes, the concentration of responses at the high end of the scale—manifesting as ceiling effects—has significant implications for the use and interpretation of the BMSLSS in research and practice. The scale's limited sensitivity at higher levels of life satisfaction may compromise its reliability and validity when assessing SWB in samples expected to have generally high satisfaction, such as in universal prevention or promotion programs. This issue is particularly relevant for intervention studies, where ceiling effects could obscure the detection of improvements among already satisfied participants.

Researchers and practitioners using the ELPI version of the BMSLSS should be aware of its psychometric limitations and exercise caution when interpreting scores at the upper end of the scale. High scores should not be automatically interpreted as indicating optimal life satisfaction, as the scale may not adequately capture nuances in SWB among highly satisfied respondents. When reporting findings, researchers should clearly acknowledge the scale's psychometric properties and discuss the potential implications for the interpretation of results. Furthermore, when selecting measures for assessing life satisfaction in Chilean early adolescents, researchers should carefully consider the psychometric properties of available instruments and their suitability for the specific research or intervention context. Indeed, given these limitations, it is advisable to adopt a multi-method approach when assessing SWB in youth populations. Using a combination of different types of scales—including both global and domain-specific measures, as well as complementary assessment methods—can provide a more comprehensive and nuanced understanding of adolescents' wellbeing (Casas, 2017). This triangulation approach helps mitigate the limitations of any single instrument and enhances the validity of research findings.

### Future directions

Future research should aim to address the limitations identified in this study and further optimize the BMSLSS for use with early adolescents in Chile and other contexts. In this regard, international initiatives such as the Children's Worlds Project have already made important strides by developing and refining domain-based instruments, including modifications to the original BMSLSS items and response formats (Casas and González-Carrasco, 2021). The Children's Worlds Domain-Based Subjective Wellbeing Scale, which incorporates adapted BMSLSS items with a 0–10 response scale, represents one such advancement that addresses some of the psychometric issues identified in traditional versions (Savahl et al., 2021). These ongoing efforts demonstrate the value of iterative scale development informed by psychometric analyses like those presented here. Future research could continue this work by exploring additional modifications.

The current study contributes to the growing body of research on measuring SWB in children and adolescents. Our findings align with previous studies that have found good overall psychometric properties of the BMSLSS using classical methods (Abubakar et al., 2016; Alfaro et al., 2015). However, our application of IRT methods allowed us to identify specific areas for improvement that were not detected in prior psychometric evaluations. This study demonstrates the value of advanced methodological approaches like IRT for refining measures of youth SWB and advancing the field of positive psychology. As the emphasis on studying and promoting SWB in young populations continues to grow, it is essential that we develop and validate precise, reliable, and sensitive measures that can accurately assess life satisfaction across different contexts and cultures.

### Limitations

It is important to acknowledge the limitations of this study. First, the findings are based on a specific adaptation of the BMSLSS for Chilean early adolescents and may not generalize to other versions or populations. Second, the study relied on cross-sectional data, which limits the ability to examine the scale's temporal stability or its sensitivity to change over time. Future research should investigate the psychometric properties of the BMSLSS using longitudinal designs to assess its utility for tracking changes in life satisfaction during this developmental period.

## Conclusions

In conclusion, this study makes significant contributions to the measurement of life satisfaction in early adolescents by conducting the first IRT analysis of the BMSLSS. Our findings underscore the importance of applying rigorous psychometric methods to evaluate and optimize measures of youth SWB. The limitations we identified in the response scale of the ELPI version of the BMSLSS highlight the need for continued research to refine the scale and improve its ability to assess life satisfaction across the full spectrum of the construct. By advancing the methodological quality of life satisfaction measures, we can enhance our understanding of youth SWB and ultimately inform interventions and policies aimed at promoting positive development in children and adolescents.

## Data Availability

Publicly available datasets were analyzed in this study. This data can be found here: https://observatorio.ministeriodesarrollosocial.gob.cl/.
