# Supplementary material for: Psychometric evaluation of the Brief Multidimensional Students' Life Satisfaction Scale (BMSLSS) in Chilean early adolescents: an Item Response Theory analysis
Source: Front Psychol. 2025 Sep 17;16:1638017. doi: 10.3389/fpsyg.2025.1638017 (PMC12486309; doi:10.3389/fpsyg.2025.1638017)
Supplement: Supplementary file 1 [file Data_Sheet_1.pdf]

Supplementary Material:

Original Brief Multidimensional Students' Life Satisfaction Scale (BMSLSS) items

and Spanish translation used in ELPI

Original BMSLSS items	Spanish translation used in ELPI
How satisfied are you with…	*¿Qué tan satisfecho(a) estás con…*
1. Your family life?	1. tu vida familiar?
2. Your friendships?	2. tus amigos y amigas?
3. Your school experience?	3. tu experiencia en el colegio?
4. Yourself?	4. contigo mismo(a)?
5. Where you live?	5. con el barrio donde vives?
